# Biventricular Impella Support for Ventricular Tachycardia Ablation in a Patient With Severe Dilated Cardiomyopathy

**DOI:** 10.1002/ccr3.72320

**Published:** 2026-03-25

**Authors:** Madeleine Tydecks, Theresa Reiter, Gabriele Hessling, Isabel Deisenhofer, Mohammad Al Fayad

**Affiliations:** ^1^ Department of Electrophysiology TUM University Hospital German Heart Center Munich Germany

**Keywords:** biventricular mechanical circulatory support, heart failure, Impella, ventricular tachycardia ablation

## Abstract

Ventricular tachycardia (VT) ablation in patients with severely impaired left ventricular (LV) function remains challenging because mapping often requires VT induction or sustained arrhythmia that may not be tolerated. Temporary mechanical circulatory support (tMCS) can improve procedural stability in high‐risk patients. We report a successful VT ablation in a 73‐year‐old man with non‐ischemic dilated cardiomyopathy, severe biventricular failure, and recurrent VT, performed under fully percutaneous biventricular support using Impella CP and Impella RP (Abiomed) via transfemoral access. Hemodynamic stability was achieved only after the addition of right‐sided support. The procedure reduced VT burden without device‐ or access‐related complications.

## Key Clinical Message

Percutaneous biventricular Impella support (Impella CP plus Impella RP, Abiomed) can provide rapid, minimally invasive hemodynamic stabilization during high‐risk VT ablation in patients with severe biventricular failure. This strategy may enable mapping/ablation otherwise not tolerated, but should be balanced against resource use and limited long‐term outcome data.

## Graphical abstract

VT ablation with percutaneous biventricular Impella support in a patients with non‐ischemic dilated cardiomyopathy and severe biventricular failure. Segmented Inheart CT, angulation a.p. (a) and LAO (c). Segmentation revealed the prior ablation site (blue arrow) and extensive intramural fibrotic tissue (asterisk). (b) fluoroscopic view of the ablation catheter position in a.p. (d) intracardiac 3D map with ablation sites, matching the intramural fibrotic tissue.

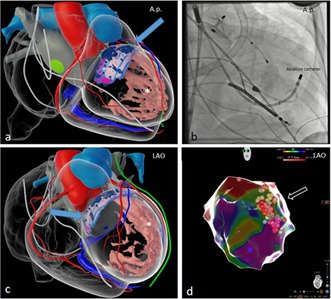

## 1 Introduction

Ventricular tachycardia (VT) in patients with structural heart disease frequently causes severe hemodynamic compromise. Catheter ablation is an established therapy for recurrent symptomatic VT despite antiarrhythmic medication and is often facilitated by VT induction/maintenance to define critical sites for mapping and termination [1]. However, patients with advanced heart failure are particularly vulnerable to acute hemodynamic decompensation (AHD) during VT ablation, which is associated with worse outcomes [2, 3].

Temporary mechanical circulatory support (tMCS) has been used in selected high‐risk VT ablations to preserve organ perfusion and enable mapping and ablation [4]. While percutaneous LV support can improve stability in many cases, isolated LV unloading may be insufficient in patients with significant right ventricular (RV) dysfunction or severe tricuspid regurgitation, where RV–LV flow mismatch can persist or worsen despite LV support [5]. Evidence for fully percutaneous biventricular microaxial support during VT ablation remains limited [6, 7]. We report a successful VT ablation performed under transfemoral biventricular Impella support (Impella CP and Impella RP) in end‐stage non‐ischemic dilated cardiomyopathy with severe biventricular failure.

## 2 History of Presentation

A 73‐year‐old man with non‐ischemic dilated cardiomyopathy and severe heart failure with reduced ejection fraction (HFrEF) (LV ejection fraction 18%, LV end‐diastolic diameter 72 mm) and a history of recurrent non‐sustained and sustained VT with syncope was admitted for VT ablation.

**FIGURE 1:**
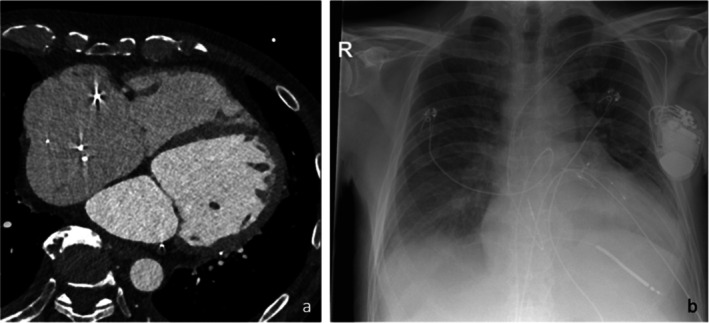
(a) CT scan 10/24 shows massive RA and biventricular dilatation. (b) Chest x‐ray (p.a.) at admission shows pleural effusions and pulmonary volume overload.

At admission, the patient showed advanced congestion with pleural effusions, ascites, and acute kidney injury on chronic kidney disease (Figure 1). Echocardiography demonstrated moderate‐to‐severe RV dysfunction (RV basal diameter 54 mm; tricuspid annular plane systolic excursion (TAPSE) 9 mm) and severe tricuspid regurgitation. Device interrogation (CRT‐D) and Holter monitoring revealed recurrent VTs (cycle length 310–340 ms) requiring antitachycardia pacing. Twelve‐lead ECG and Holter also demonstrated frequent premature ventricular complexes (PVCs), with the predominant morphology suggesting an anterior/subaortic LV origin (Table 1).

**TABLE 1:** Clinical timeline.

Time	Event
2015	Diagnosis of DCM/recurrent HF decompensations
2017	CRT‐D implantation; persistent AF diagnosed
2020	PCI for two‐vessel CAD
2022	LAA occluder implantation
~6 months before index admission	Planned PVC ablation with LV support aborted due to hemodynamic compromise; CT segmentation performed
02/2025	Index admission with decompensated HF and recurrent VT
14 March 2025	VT ablation under biventricular Impella support
Discharge (6 days post ablation)	Reduced nsVT burden; amiodarone initiated
4 weeks post ablation	One brief nsVT (7 beats); no ICD therapies
3 months post ablation	Death due to terminal heart failure

The patient had long‐standing dilated cardiomyopathy with recurrent decompensation. A CRT‐D system was implanted in 2017. Four weeks prior to the index admission, the patient received intravenous levosimendan for severe heart failure.

Two‐vessel coronary artery disease was treated in 2020, and progression was excluded by invasive assessment during the index hospitalization. Persistent atrial fibrillation was diagnosed in 2017; the patient underwent multiple ablations in both atria. In 2022, a left atrial appendage (LAA) occluder was implanted. Six months prior to the index admission, ablation of polymorphic PVCs via transseptal LV access with LV mechanical support was planned. Prior to the procedure, a high‐resolution late‐enhancement cardiac CT was obtained and externally segmented (InHeart Medical, Pessac, France), showing anterior and lateral intramural fibrosis (Figure 3) [8]. The procedure was aborted due to hemodynamic compromise despite LV‐only mechanical support.

## 3 Investigations and Treatment

Before ablation, hemodynamic optimization with intravenous diuretics and short‐term levosimendan was performed. A PAINESD risk score of 12 (age > 60 years, NYHA class IV, EF < 25%) indicated a moderate‐to‐high risk of AHD during VT ablation [3].

Vascular access was evaluated by ultrasound prior to the procedure. The procedure was performed under deep sedation using propofol. Hemodynamic monitoring included invasive arterial blood pressure measurement, pulse oximetry, and blood gas analysis.

Impella CP (Abiomed, LV support) was inserted via the left common femoral artery using a 14F arterial sheath under ultrasound‐guided puncture and fluoroscopic guidance. During the procedure, Impella CP was operated at P‐9, providing an estimated flow of 3.8 L/min (console range 3.3–4.3 L/min). Console‐derived pressure tracings at the documented time point showed an aortic pressure of 85/59 mmHg (mean 67 mmHg) and a LV pressure of 97/3 mmHg. Purge parameters were stable (purge flow 15.7 mL/h, purge pressure 440 mmHg).

**FIGURE 2:**
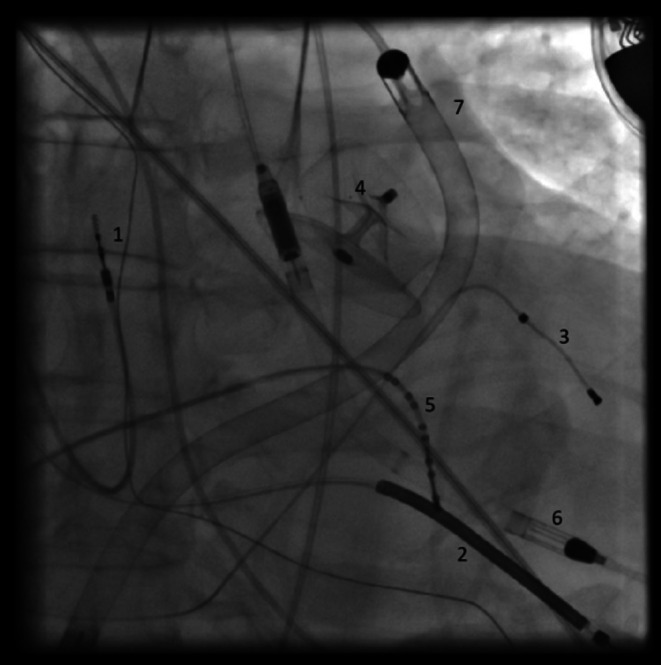
AP fluoroscopic view showing: RA pacing electrode (1), RV shock coil (2), CS pacing electrode (3), LAA occluder (4), RV diagnostic catheter (5), Impella CP (6), and Impella RP (7). Impella CP is positioned across the aortic valve with the inlet in the LV and the outlet in the ascending aorta; Impella RP is positioned from the IVC/RA through the RV into the pulmonary artery.

Impella RP (Abiomed, RV support) was inserted via the right common femoral vein using a 23F venous sheath and advanced under fluoroscopic guidance to the pulmonary artery position (Figure 2). The Impella RP was operated at P‐7, providing an estimated flow of 3.5 L/min (console range 3.2–3.7 L/min). Purge parameters were stable (purge flow 11.2 mL/h, purge pressure 493 mmHg). Both parameters displayed on the monitors of the two Impella devices were recorded simultaneously.

No suction events, device alarms, or need for repositioning occurred, and there was no evidence of clinically relevant hemolysis.

For electrophysiology catheters and mapping, additional vascular access was obtained. A right common femoral arterial 8F sheath was used for retrograde LV mapping. Additional venous sheaths (approximately 8F) were used for standard diagnostic catheters (right ventricle/coronary sinus), as required.

Both Impella devices were initiated prior to electroanatomic mapping. Total support duration was 240 min for Impella CP and 210 min for Impella RP. Both devices were removed immediately at the end of the procedure in the electrophysiology laboratory (Table 2).

**TABLE 2:** Periprocedural hemodynamics and support parameters.

Time point	RR/MAP	Vasopressors	Impella CP (P/L·min^−1^)	Impella RP (P/L·min^−1^)	Notes
Baseline (after induction, pre‐MCS)	66/40 mmHg (MAP 49 mmHg)	Norepinephrine + etilefrine	—	—	
After CP placement	75/52 mmHg (MAP 60 mmHg)	Norepinephrine + etilefrine	P‐9/3.8 (3.3–4.3)	—	Persistent hypotension
After RP placement	96/78 mmHg (MAP 84 mmHg)	Norepinephrine + etilefrine	P‐9/3.8	P‐7/3.5 (3.2–3.7)	Stabilization achieved
BP drop (~2 h)	80/57 mmHg (MAP 65 mmHg) → 96/84 mmHg (MAP 88 mmHg)	Norepinephrine + etilefrine	P‐9/3.8	P‐7/3.5	Resolved after intravenous fluids
End of procedure	84/54 mmHg (MAP 64 mmHg)	Continued	Removed	Removed	Removed in EP lab

Periprocedural anticoagulation was achieved with unfractionated heparin (initial bolus 7000 IE) targeting an activated clotting time (ACT) of 350–400 s. ACT was assessed repeatedly during the procedure every 20 min (measured ACT range 380). A heparinized purge solution was used for both Impella systems.

At baseline under deep sedation prior to mechanical support, the invasive arterial pressure was 66/40 mmHg (MAP 49 mmHg), requiring vasoactive support with norepinephrine and etilefrine. After the initiation of left‐sided support with Impella CP, the patient remained hemodynamically unstable with hypotension (75/52 mmHg (MAP 60 mmHg)), Hemodynamic stabilization was achieved only after the addition of right‐sided support with Impella RP, resulting in subsequent arterial pressure of 96/78 mmHg (MAP 84 mmHg).

Approximately 2 h after initiation of mechanical support, a transient blood pressure drop to 80/57 mmHg (MAP 65 mmHg) occurred and resolved after intravenous fluid substitution, restoring arterial pressure to 96/84 mmHg (MAP 88 mmHg).

LV electroanatomic mapping was performed using a retrograde aortic approach via a right femoral arterial 8F sheath. A retrograde strategy was chosen to avoid additional transseptal instrumentation in the setting of a large‐bore right femoral venous access for Impella RP and because the anticipated target region (dominant PVC/VT morphology suggesting an anterior/subaortic LV origin) could be reached reliably without added benefit from transseptal puncture.

Mapping was performed with CARTO3 (Biosense Webster) and a ThermoCool ST/SF catheter (Biosense Webster). Voltage mapping and electrogram annotation demonstrated an anterolateral scar region with fractionated electrograms and late potentials. Due to the patient's limited hemodynamic tolerance, no programmed VT induction was performed; ablation was guided by a substrate‐based strategy combined with pace mapping targeting the anterolateral substrate. During RF delivery, the clinical VT/PVC morphology occurred transiently and terminated during ablation, supporting the selected target region.

**FIGURE 3:**
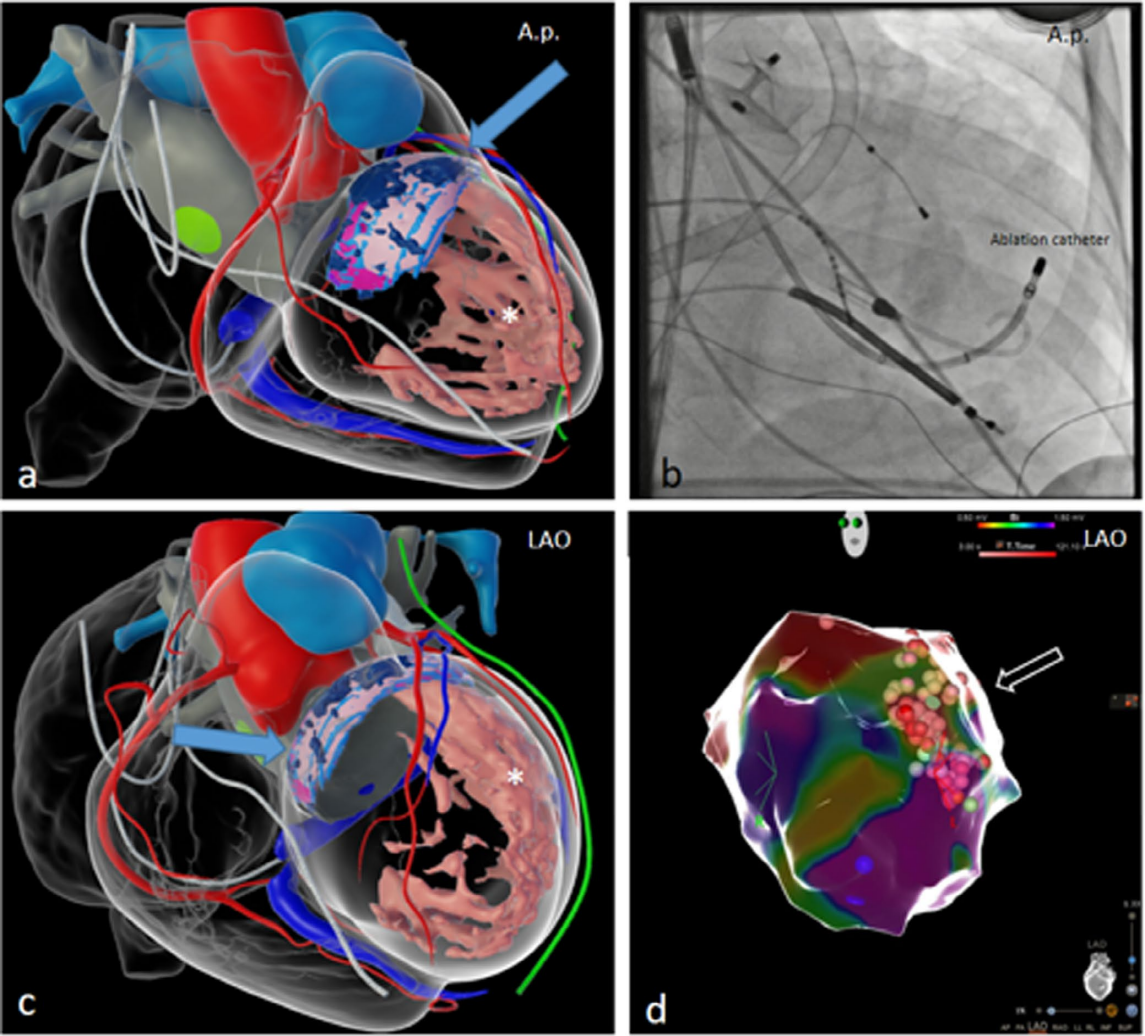
Inheart CT scans, fluoroscopy and intracardiac 3D map. Segmented Inheart CT, angulation a.p. (a) and LAO (c). Segmentation revealed the prior ablation site (blue arrow) and extensive intramural fibrotic tissue (asterisk). (b) fluoroscopic view of the ablation catheter position in a.p. (d) intracardiac 3D map with ablation sites, matching the intramural fibrotic tissue.

The previously obtained high‐resolution late‐enhancement cardiac CT was segmented externally (InHeart Medical, Pessac, France). The resulting 3D CT model was merged with the CARTO3 geometry and used to guide substrate targeting. CT‐defined anterolateral intramural fibrosis informed the selection of ablation targets, with lesions being preferentially delivered along scar border zones that matched areas with fractionated electrograms/late potentials on the electroanatomic map (Figure 3). At the end of the procedure, echocardiography ruled out pericardial effusion. The left femoral arterial access was closed using two AngioSeal devices (6F and 8F) with no vascular complications. The venous large‐bore access was closed with a figure‐of‐eight (U‐) suture without complications.

## 4 Outcome and Follow‐Up

After ablation, the patient was monitored in the intensive care unit for three days; mobilization started 24 h after the procedure. Post‐interventional Holter ECG and CRT‐D interrogation showed markedly fewer non‐sustained VT episodes. Amiodarone therapy was initiated under monitoring to support rhythm stability during the myocardial healing period.

Post‐procedurally, intravenous unfractionated heparin was continued for 6 h, followed by aspirin 100 mg once daily, considering the patient's history of atrial fibrillation and prior LAA occluder.

The patient was discharged six days after ablation. The patient was seen 4 weeks after the procedure, and device interrogation and Holter monitoring demonstrated only one non‐sustained VT episode of 7 beats without syncope and the known persistent atrial fibrillation. There were no ICD therapies and no evidence of recurrent VT storm. The patient died 3 months after ablation due to progressive terminal heart failure.

## 5 Discussion

This case report presents the successful application of minimally invasive, periinterventional biventricular Impella support during VT ablation in a complex patient with dilated cardiomyopathy and severely reduced biventricular function.

Several studies have demonstrated the benefit of biventricular MCS in patients suffering from cardiogenic shock [7]. Data on the transfer of this biventricular approach for VT ablation are still very limited [5]. In patients with globally reduced heart function, the application of a single LV MCS might not be successful in maintaining hemodynamic stability. Isolated LV support might paradoxically increase RV afterload and potentially lead to RV failure and hemodynamic collapse (Box 1). By unloading the LV, Impella CP reduces LV wall stress, improves coronary perfusion, and enhances LV contractility, sometimes associated with a transient pressure drop. Impella RP supports RV function, reducing RV volume overload, improving pulmonary blood flow, and mitigating the risk of RV failure [5, 9]. These effects can cause a transient increase in volume demand that reflects the increased blood circulation due to increased biventricular blood flow as well as the prolonged intervention time, the effects of deep sedation, and the potential for impaired lusitropy following extensive ablation [10, 11].

Unlike prior cases, no surgical access for the pumps was chosen [3]. The minimally invasive implantation strategy offers significant advantages over surgically implanted MCS for severely impaired patients. It allows for faster deployment and removal of the devices, simplified vascular closure, and enhanced mobilization.

While further research is warranted to evaluate the long‐term outcomes and cost‐effectiveness of this approach in a larger patient population, this case suggests that biventricular Impella MCS support may represent a valuable tool in the armamentarium of the electrophysiologist managing complex VT ablation procedures in patients with highly impaired ventricular function.

AHD is a major limitation of VT ablation in advanced cardiomyopathy and has been associated with increased mortality, motivating risk stratification and protected VT ablation concepts [2, 3]. Tools such as the PAINESD score help identify patients at higher risk of periprocedural deterioration [3]. Observational analyses suggest that prophylactic percutaneous LV support in selected high‐risk patients may reduce AHD and death/transplant during follow‐up, though effects on VT‐free survival are less consistent and must be weighed against complications and resource utilization [4].

In patients with concomitant RV dysfunction, severe tricuspid regurgitation, or systemic congestion, isolated LV unloading may not translate into adequate systemic forward flow. RV–LV coupling can remain impaired, and paradoxical hemodynamic deterioration may occur if LV output is augmented without sufficient RV throughput [5]. Our patient had advanced RV dysfunction and severe TR, and stabilization occurred only after addition of Impella RP at therapeutic flows, supporting the concept that planned biventricular support should be considered when RV reserve is limited and prior LV‐only strategies failed.

Alternative tMCS options include VA‐ECMO (with or without LV unloading) [12], left atrial–arterial bypass systems (e.g., TandemHeart) [13], and dedicated RV support strategies (e.g., ProtekDuo‐based RVAD) [14]. VA‐ECMO provides oxygenation and robust circulatory support but can increase LV afterload and may require unloading/venting strategies [12]. TandemHeart offers high flows but requires transseptal cannulation and a circuit‐based approach [13]. ProtekDuo provides percutaneous RV support with distinct cannulation and management considerations [14]. In contrast, a fully percutaneous microaxial “Bi‐Pella” approach can be rapidly deployed and removed and avoids surgical cutdown, but requires meticulous monitoring for vascular complications and hemolysis and remains supported by limited VT‐ablation‐specific data [6, 7].

BOX 1 **Pragmatic escalation concept to RV support during protected VT ablation [2, 3, 15].**

Consider planned biventricular support if ≥ 2 are present: TAPSE ≤ 10 mm, severe tricuspid regurgitation, RV basal diameter ≥ 50 mm, severe systemic congestion/ascites, marked renal dysfunction, or prior intolerance/failure of LV‐only support during VT ablation.

Escalate to RV support intra‐procedurally if, despite adequate LV unloading, persistent MAP < 65 mmHg, rising lactate, escalating vasopressor requirement, echocardiographic signs of RV distension/poor forward flow, or clinical evidence of RV–LV mismatch occurs.

Document safety/performance metrics (flows, vasoactive doses, ACT targets, suction/alarms, hemolysis, and access‐site outcomes) to enable reproducibility and benchmarking.

## 6 Limitations and Ethical Considerations

This report is limited by its single‐patient design and the patient's terminal baseline heart failure, constraining generalizability and interpretation of long‐term benefit. Although arrhythmia burden decreased, overall survival was driven by progressive end‐stage cardiomyopathy. High‐resource interventions in terminal patients require careful selection, shared decision‐making, and alignment with goals of care. Larger studies are needed to define optimal selection and compare biventricular microaxial support with alternative tMCS strategies in VT ablation.

## Author Contributions

**Madeleine Tydecks:** conceptualization, investigation, visualization, writing – original draft. **Theresa Reiter:** conceptualization, visualization, writing – original draft, writing – review and editing. **Gabriele Hessling:** methodology, supervision, writing – review and editing. **Isabel Deisenhofer:** supervision, validation, writing – review and editing. **Mohammad Al Fayad:** investigation, visualization, writing – review and editing.

## Funding

The authors have nothing to report.

## Ethics Statement

Institutional review board approval was not required according to local regulations for this single‐patient case report.

## Consent

Written informed consent was obtained from the patient to publish this report in accordance with the journal's patient consent policy.

## Conflicts of Interest

The authors declare no conflicts of interest.

## Data Availability

The data that support the findings of this study are available on request from the corresponding author. The data are not publicly available due to privacy or ethical restrictions.
